# A clinical decision support tool for improving adherence to guidelines on anticoagulant therapy in patients with atrial fibrillation at risk of stroke: A cluster-randomized trial in a Swedish primary care setting (the CDS-AF study)

**DOI:** 10.1371/journal.pmed.1002528

**Published:** 2018-03-13

**Authors:** Lars O. Karlsson, Staffan Nilsson, Magnus Bång, Lennart Nilsson, Emmanouil Charitakis, Magnus Janzon

**Affiliations:** 1 Department of Cardiology, and Department of Medical and Health Sciences, Linköping University, Linköping, Sweden; 2 Primary Health Care Center in Vikbolandet, and Department of Medical and Health Sciences, Linköping University, Vikbolandet, Sweden; 3 Department of Computer and Information Science, Linköping University, Linköping, Sweden; Scripps Translational Science Institute, UNITED STATES

## Abstract

**Background:**

Atrial fibrillation (AF) is associated with substantial morbidity, in particular stroke. Despite good evidence for the reduction of stroke risk with anticoagulant therapy, there remains significant undertreatment. The main aim of the current study was to investigate whether a clinical decision support tool (CDS) for stroke prevention integrated in the electronic health record could improve adherence to guidelines for stroke prevention in patients with AF.

**Methods and findings:**

We conducted a cluster-randomized trial where all 43 primary care clinics in the county of Östergötland, Sweden (population 444,347), were randomized to be part of the CDS intervention or to serve as controls. The CDS produced an alert for physicians responsible for patients with AF and at increased risk for thromboembolism (according to the CHA_2_DS_2_-VASc algorithm) without anticoagulant therapy. The primary endpoint was adherence to guidelines after 1 year. After randomization, there were 22 and 21 primary care clinics in the CDS and control groups, respectively. There were no significant differences in baseline adherence to guidelines regarding anticoagulant therapy between the 2 groups (CDS group 70.3% [5,186/7,370; 95% CI 62.9%–77.7%], control group 70.0% [4,187/6,009; 95% CI 60.4%–79.6%], *p* = 0.83). After 12 months, analysis with linear regression with adjustment for primary care clinic size and adherence to guidelines at baseline revealed a significant increase in guideline adherence in the CDS (73.0%, 95% CI 64.6%–81.4%) versus the control group (71.2%, 95% CI 60.8%–81.6%, *p* = 0.013, with a treatment effect estimate of 0.016 [95% CI 0.003–0.028]; number of patients with AF included in the final analysis 8,292 and 6,508 in the CDS and control group, respectively). Over the study period, there was no difference in the incidence of stroke, transient ischemic attack, or systemic thromboembolism in the CDS group versus the control group (49 [95% CI 43–55] per 1,000 patients with AF in the CDS group compared to 47 [95% CI 39–55] per 1,000 patients with AF in the control group, *p* = 0.64). Regarding safety, the CDS group had a lower incidence of significant bleeding, with events in 12 (95% CI 9–15) per 1,000 patients with AF compared to 16 (95% CI 12–20) per 1,000 patients with AF in the control group (*p* = 0.04). Limitations of the study design include that the analysis was carried out in a catchment area with a high baseline adherence rate, and issues regarding reproducibility to other regions.

**Conclusions:**

The present study demonstrates that a CDS can increase guideline adherence for anticoagulant therapy in patients with AF. Even though the observed difference was small, this is the first randomized study to our knowledge indicating beneficial effects with a CDS in patients with AF.

**Trial registration:**

ClinicalTrials.gov NCT02635685

## Abstract 2

In a cluster randomized trial, Lars Karlsson and colleagues examine the effectiveness of a clinical decision support tool for improving adherence to guidelines on anticoagulant therapy in patients with atrial fibrillation.

## Author summary

**Why was this study done?:**

- Atrial fibrillation is the most common heart arrhythmia, affecting approximately 3% of the population.
- In combination with certain risk factors, atrial fibrillation carries an increased risk of stroke.
- Despite evidence that we can reduce this risk with the use of blood thinners, underuse remains significant worldwide.

**What did the researchers do and find?:**

- We developed an automated clinical decision support tool integrated in the electronic health record.
- The clinical decision support tool produced an alert (pop-up) for patients with atrial fibrillation at risk of stroke without appropriate treatment.
- We were able to show that this straightforward intervention increased the usage of blood thinners in patients with atrial fibrillation.

**What do these findings mean?:**

- The current findings show that an easy-to-implement intervention can improve patient care for patients with atrial fibrillation at risk for stroke.
- This study highlights the possibility of using available medical record data in an automated fashion.
- The results encourage further investigation of clinical decision support tools within other areas of medicine.

## Introduction

Atrial fibrillation (AF) has a prevalence of more than 3% in developed countries [1] and is estimated to affect more than 33 million people worldwide [2]. The condition carries an increased risk of thromboembolism, in particular stroke. The Global Burden of Disease project estimated the incidence of ischemic stroke at more than 6 million cases worldwide in 2013 [3]. Of these, approximately 20% are attributable to AF [3,4]. Moreover, stroke in patients with AF more often leads to permanent disability and death than stroke from other causes [5].

Numerous studies have shown that the risk for ischemic stroke can be reduced by approximately 60%–70% with the use of anticoagulant therapy in patients with additional risk factors for stroke and concurrent AF [6]. The European Society of Cardiology recommends using the CHA_2_DS_2_-VASc algorithm to identify patients at increased risk for stroke in whom anticoagulant therapy should be considered [7,8]. Anticoagulant agents recommended include warfarin and non–vitamin K oral anticoagulants (NOACs).

Despite the recommendations in current guidelines, there remains substantial undertreatment in this group of patients [9–11]. Moreover, in patients presenting with ischemic stroke, more than 4 out of 5 patients had inadequate therapeutic anticoagulation preceding the ischemic event, further highlighting the clinical importance of adequate treatment [12]. The reasons for anticoagulant underuse are most likely manifold, including difficulties in identifying AF and the conditions constituting the CHA_2_DS_2_-VASc algorithm. Furthermore, there is a reluctance to use potent anticoagulant drugs due to increased risks of falling and bleeding. Finally, a recent report demonstrated that underuse is more pronounced in middle- and low-income countries, indicating that patients’ and physicians’ awareness as well as educational levels may contribute to the inequality regarding anticoagulant use [13].

Clinical decision support systems are receiving increased attention as tools to reduce costs and improve care. Reports indicate a promising potential, but evidence with regards to clinical outcome is conflicting [14–16], with systematic reviews reporting only modest improvements of the healthcare process [14–18]. Of note, a recent review could not establish a beneficial effect on mortality with this technology [18]. Furthermore, in the case of anticoagulant therapy in AF, recent reports did not show any beneficial effects of 2 different clinical decision support systems in primary care [19,20].

The clinical decision support tool (CDS) for stroke prevention under investigation in this study is a computerized instrument developed by cardiologists and primary care physicians in collaboration with Cambio Healthcare Systems. This CDS is integrated in the ordinary electronic health record (EHR) and uses medical record data to identify patients with a diagnosis of AF and 1 or more risk factors according to the CHA_2_DS_2_-VASc algorithm who have not been prescribed anticoagulant therapy.

The aim of the present study was to investigate if a clinical decision support system could increase adherence to guidelines regarding stroke prevention in patients with AF in a cluster-randomized trial in the primary care setting.

## Methods

The technical aspects of the CDS and the study design, with additional secondary outcomes, have been published elsewhere [21]. What follows is an overview of the CDS and the overall study design.

### The clinical decision support tool

**Fig 1:**
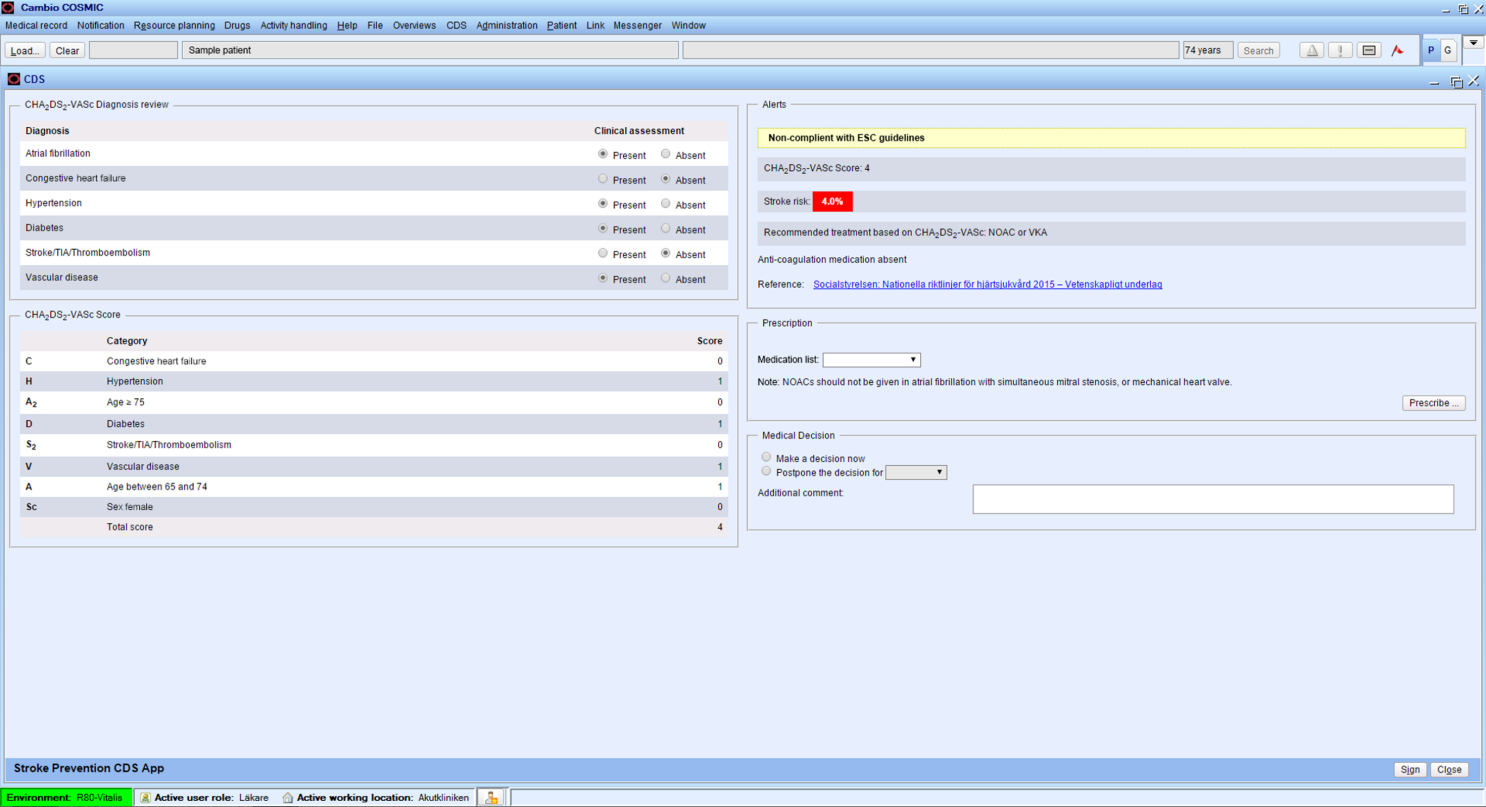
The clinical decision support tool for improving adherence to guidelines on anticoagulant therapy. CDS, clinical decision support tool; NOAC, non–vitamin K oral anticoagulant; VKA, vitamin K antagonist; TIA, transient ischemic attack.

The CDS was developed in a collaboration between Cambio Healthcare Systems (the supplier of the EHRs in the county of Östergötland), the Department of Cardiology at Linköping University Hospital, and primary care professionals. The CDS is activated when a patient with a diagnosis of AF or atrial flutter (International Classification of Diseases [ICD] code I48) is being logged into the EHR. If the patient has additional risk factors for stroke according to the CHA_2_DS_2_-VASc algorithm (based on age, sex, and ICD codes listed in S1 Text) and is without anticoagulant therapy according to the medication list, a pop-up screen warning appears (Fig 1). By clicking on the pop-up, the responsible physician is directed to an overview of the patient’s risk factors including a calculated annual risk for stroke for the patient and a recommendation to consider anticoagulant therapy. The physician can thereafter decide to prescribe anticoagulant therapy in accordance with the current guidelines (warfarin and the NOACs are listed in alphabetic order) or, alternatively, postpone the decision or make a decision to refrain from medication. If the choice is made to refrain from medication, the physician is asked to choose from a set of predetermined alternative reasons in order to monitor the main reason for not adhering to the guidelines. Regardless of the decision made, a note in the EHR is automatically generated. If the patient already has anticoagulant therapy in accordance with current guidelines, no screen warning appears.

### Study population

CDS-AF (Clinical Decision Support for Stroke Prevention in Atrial Fibrillation) (ClinicalTrials.gov registration number NCT02635685) was a cluster-randomized trial performed in the primary care setting. The study was conducted in the county of Östergötland, situated in the southeast of Sweden. The catchment area had a total population of 444,347 inhabitants at study initiation, of which 14,134 had a diagnosis of AF. In Sweden, the healthcare system is organized in a way that each individual in need of primary healthcare resources is asked to choose a specific primary care clinic. In case medical attention is needed, the patient preferably visits the chosen clinic, unless emergency or specialized care is required. Typically, anticoagulant therapy for AF is initiated at primary care clinics. Sweden has a publicly funded healthcare system. As such, no reimbursements are provided for individual healthcare providers or primary care clinics regarding anticoagulant therapy.

Data from 2015 indicated that 70% of the patients with AF in the county of Östergötland were adherent to guidelines regarding anticoagulant therapy at study initiation. Considering an increase in adherence from 70% to 75% in the intervention group clinically meaningful, a sample size calculation for a randomized trial with a fixed number of clusters was performed [22]. Assuming an intraclass correlation coefficient of 0.01, a sample size of 2,494 patients would give a power of 80% (alpha 0.05) to detect a significant difference between the intervention and control group. However, our primary aim was to include the whole population of the catchment area.

All primary care clinics in the county of Östergötland using the Cambio EHR were eligible for participation in the study. At the time of inclusion, all primary care clinics—all of which were using the Cambio EHR—were approached for participation and randomized to intervention with the CDS or to serve as controls (i.e., continue with usual care). The clinics were stratified into 4 strata based on the number of patients listed at each clinic and current adherence to guidelines (i.e., the percentage of patients with a diagnosis of AF and CHA_2_DS_2_-VASc ≥ 1, but not including those with CHA_2_DS_2_-VASc = 1 based on female sex, who were currently prescribed anticoagulant therapy) [7]. The majority of patients with a diagnosis of AF were identified before study initiation, but individuals with a new diagnosis of AF during the study period were also included in the final analysis.

After stratification, the clinics were randomized 1:1 to CDS intervention or control. Forum Östergötland, a core facility with statistical expertise at Linköping University, Sweden, performed the randomization sequence. Before study initiation, all primary care clinics received general information regarding AF and the indication for anticoagulant therapy in this setting. The clinics randomized to intervention with CDS received a briefing regarding the technical aspects of the CDS, and this information was thereafter available to the physicians on the local intranet throughout the study period. The primary care physicians were therefore not blinded to group assignment, but the patients and the persons responsible for extracting data from the EHR were unaware of group allocation.

The main phase of the study lasted for 12 months, with study initiation on 11 January 2016.

### Informed consent and ethical considerations

The primary research subjects in this study were the primary care physicians who were randomized to receive or not receive the CDS intervention. The population of physicians and the primary healthcare clinics are heterogeneous groups. They represent the real-world setting in which anticoagulants are being prescribed. To receive a true picture of the feasibility and effectiveness of the CDS in supporting prescription of anticoagulants, we considered it important to cover all primary healthcare clinics and, if possible, all physicians working in the catchment area. According to Swedish laws and ethics regulations in human research, it is acceptable to perform this type of research without formal informed consent from each participant, if the consent procedure could be expected to severely limit the study realization or the quality and generalizability of the data obtained from the study. In this study, all physicians received oral and written information about the study, but no formal informed consent was collected from each study participant. A resource certification was, however, obtained from the head of each primary care clinic participating in the study.

The patients receiving or not receiving anticoagulants were the secondary research subjects in this study. Patients expect their physicians to use the best available knowledge in order to make good clinical decisions. In this regard, physicians can employ various written guidelines and recommendations. We considered that asking the patients whether their physician should use a CDS would be of questionable benefit from a patient perspective. In addition, there was an obvious risk that the included population would be non-representative for the real-world population. Thus, we decided not to obtain any formal informed consent from the patients. The patients were verbally informed about the study by their physician, as found appropriate.

The Regional Ethical Review Board of Linköping found the study to be in accordance with Swedish laws and regulations for ethical approval in human research and approved the study (Dnr: 2015/204-31). The study was conducted in accordance with the principles in the Declaration of Helsinki.

### Outcomes

#### Primary outcome

The primary endpoint was the proportion of patients eligible for stroke prophylaxis who were prescribed anticoagulant therapy 12 months after study initiation. Data were extracted from the EHR system at baseline and at study closure, and the results presented at the cluster level. At these 2 time points, current adherence to guidelines was calculated (the percentage of patients with a diagnosis of AF and CHA_2_DS_2_-VASc ≥ 1 [but not including those with CHA_2_DS_2_-VASc = 1 based on female sex] who were currently prescribed anticoagulant therapy, i.e., had an active prescription in the EHR medication list for warfarin, apixaban, dabigatran, or rivaroxaban; ATC codes as per S1 Text).

#### Secondary outcomes

To investigate whether a clinical decision support system could reduce the incidence of stroke, TIA, and systemic thromboembolism compared to standard care, data were extracted from the Cambio EHR. Diagnosis of stroke, TIA, and systemic thromboembolism (ICD codes I63–64, G45, I74) was compiled at study end (11 January 2017). In order to monitor a late effect on clinical endpoints, in future analyses we will extract data 3 and 6 years after study completion. This will be done through extraction from the National Patient Register (National Board of Health and Welfare, Sweden), a database that covers all diagnoses recorded from inpatient care in Sweden.

To investigate the reasons for deviation from guidelines regarding anticoagulant therapy in patients with AF, the responsible physician was asked to choose from 1 of 3 prespecified reasons listed in the CDS: “patient preference,” “contraindication,” or “other reason.” In the last case, the physician was requested to state a reason for not following guidelines, a mandatory measure.

Other secondary outcomes will be published separately on the usefulness, ease of use, and acceptance of the CDS as well as a health economic analysis of the approach.

#### Safety outcome

With the study not conducted on an individual level, but rather with a cluster-randomized trial design, possible adverse effects of the CDS intervention were monitored at the clinic level. A major possible adverse effect with anticoagulant therapy is an increased incidence of bleeding. In order to monitor this issue during the study period, we extracted data directly from the EHR. The log constituted the number of patients with AF who were started on anticoagulants with a diagnosis of clinically significant bleeding (ICD codes in S1 Text).

The study was monitored and assessed through an external safety board that consisted of 3 professionals not associated with the study. This board received an extract of the safety outcome from the EHR 3, 6, and 9 months after study initiation. The board could recommend discontinuing the study if there was a significant increase in bleeding events in the CDS group based on the bleeding extractions as per above.

### Data collection

The information needed to evaluate primary and secondary outcomes was calculated for each patient at the clinic level. For the most part, this information could be collected from the EHR. In future analyses, when data extraction is performed after study closure through the National Patient Register from the National Board of Health and Welfare, this will be done in a decoded manner. All results are presented at the clinic level, without the possibility of identifying individuals.

### Statistical considerations

Baseline characteristics are presented as mean with 95% CI for continuous data that are normally distributed, and as median (25th–75th percentile) for data that are not normally distributed. Categorical data are presented as counts with percentages. The primary endpoint was the proportion of patients with AF receiving anticoagulant therapy (out of the total number of patients eligible for medication) in each cluster at 12 months. The primary analysis was a linear regression of the cluster proportion of adherence to guidelines on randomized treatment, adjusted for baseline adherence to guidelines, and weighted by cluster size [23]. We also performed subgroup analysis using linear regression modeling based on the baseline stratification where we compared clinics with low and high adherence to guidelines (first and fourth quartiles), respectively. Intraclass correlation coefficients were calculated using the formula by Shrout and Fleiss [24].

The bleeding and thromboembolic events (stroke, TIA, or systemic thromboembolism) are presented as a proportion. The numerator is the total number of patients with AF initiated on oral anticoagulants after study commencement with a clinically significant bleeding or thromboembolic event since study initiation. The denominator is the total number of patients with AF initiated on anticoagulant therapy after study commencement. The chi-squared test was used at each extraction to detect possible differences in proportions of patients with a significant bleeding or thromboembolic event between the intervention and control groups.

The statistical analysis was performed with SPSS version 24.0 (SPSS Statistics for Windows, version 24.0; IBM, Armonk, NY).

### Deviation from study plan

In our initial study design, a calculation of the number of times the clinical decision support pop-up screen was shown was not included. During the study period, however, we realized that this would contribute valuable information on the use of the system and possibly add to the overall evaluation of the intervention. Therefore, this analysis was later included in the study plan.

## Results

**Fig 2:**
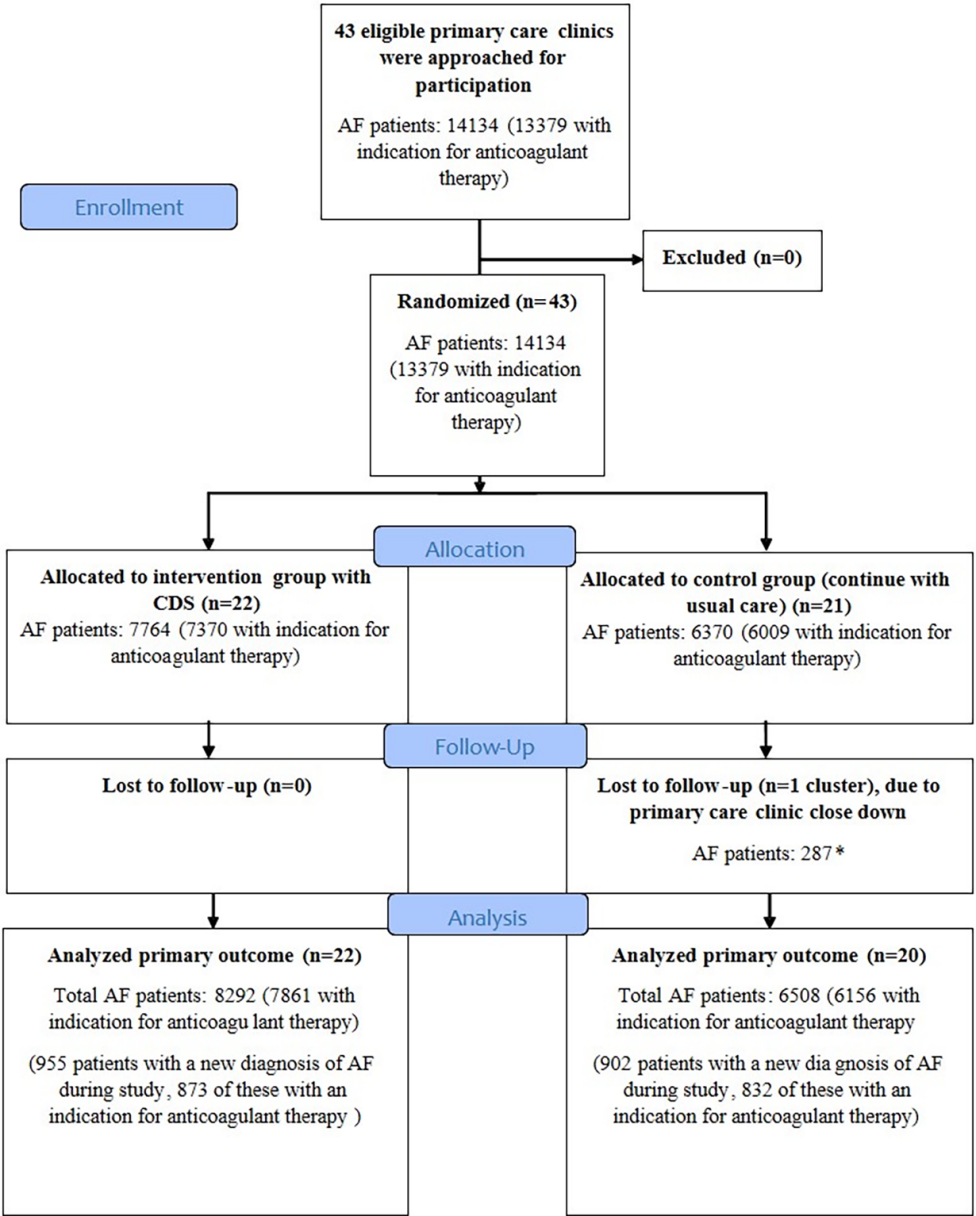
The CONSORT flow diagram of trial participants. *Patients were transferred to other primary care clinics and were included in the final intention to treat analysis. AF, atrial fibrillation; CDS, clinical decision support tool.

All 43 primary care clinics in the county of Östergötland eligible for inclusion consented to participate in the study. Of the 14,134 patients with AF at baseline, 13,379 had an indication for anticoagulant therapy according to current guidelines. One primary care clinic in the control group was lost to follow-up due to closedown. The 287 patients with AF listed at that clinic were allocated to other clinics and were included in the final intention to treat analysis. In the final analysis, 42 primary care clinics with a total of 14,800 patients with AF were included (Fig 2). During the study period, 1,857 patients received a new diagnosis of AF and were included in the final analysis.

### Baseline characteristics

Baseline characteristics for the population with AF at study commencement and the patients with a new diagnosis of AF during the study are presented in Table 1. There were no significant differences between the 2 groups at baseline.

**Table 1:** Baseline characteristics in the CDS and control groups, and patients with a new diagnosis of AF during the study, who were included in the final analysis.

Characteristic	CDS group (*n =* 22)	Control group (*n =* 21)	Patients with a new diagnosis of AF during study		
			All	CDS group only	Control group only
Total population	232,561	211,786	n/a	n/a	n/a
Total population with AF	7,764	6,370	1,857	955	902
Total population with AF and indication for anticoagulant therapy	7,370	6,009	1,705	873	832
Primary care clinic list size	10,570 ± 5,032	10,085 ± 3,658	n/a	n/a	n/a
CHA_2_DS_2_-VASc score	4 (3–5)	4 (3–5)	3 (2–4)**	3 (2–4)	3 (2–4)
Age ≥ 75 years	4,622 (59%)	3,713 (58%)	1,015 (55%)**	536 (56%)	479 (53%)
Age 65–74 years	2,092 (27%)	1,704 (27)%	544 (29%)*	262 (27%)	282 (31%)
Age < 65 years	1,050 (14%)	953 (15%)	298 (16%)*	157 (16%)	141 (16%)
Female sex	3,328 (43%)	2,740 (43%)	784 (42%)	417 (44%)	367 (41%)
Hypertension	6,082 (78%)	4,881 (77%)	1,289 (69%)**	659 (69%)	630 (70%)
Congestive heart failure	2,735 (35%)	2,270 (36%)	323 (17%)**	161 (17%)	162 (18%)
Diabetes mellitus	1,763 (23%)	1,454 (23%)	342 (18%)**	174 (18%)	168 (19%)
Vascular disease	2,630 (34%)	2,122 (33%)	356 (19%)**	182 (19%)	174 (19%)
Chronic kidney disease	308 (4%)	228 (4%)	52 (3%)*	27 (3%)	25 (3%)
Stroke, TIA, or systemic thromboembolism	1,435 (18%)	1,165 (18%)	260 (14%)**	130 (14%)	130 (14%)

Values are presented as mean ± SD, *n* (%), or median (25th–75th percentile). The study period was 11 January 2016 to 11 January 2017. There were no significant differences in baseline characteristics between the CDS and control groups regarding the population included at baseline or in the patients with a new diagnosis of AF during the study. ICD codes constituting the different diagnosis are listed in S1 Text.

*p < 0.05 versus the overall baseline population (CDS and control group analyzed together).

**p < 0.01 versus the overall baseline population (CDS and control group analyzed together).

AF, atrial fibrillation; CDS, clinical decision support tool; n/a, not applicable; TIA, transient ischemic attack.

### Proportion of eligible patients prescribed anticoagulant therapy after 12 months

**Fig 3:**
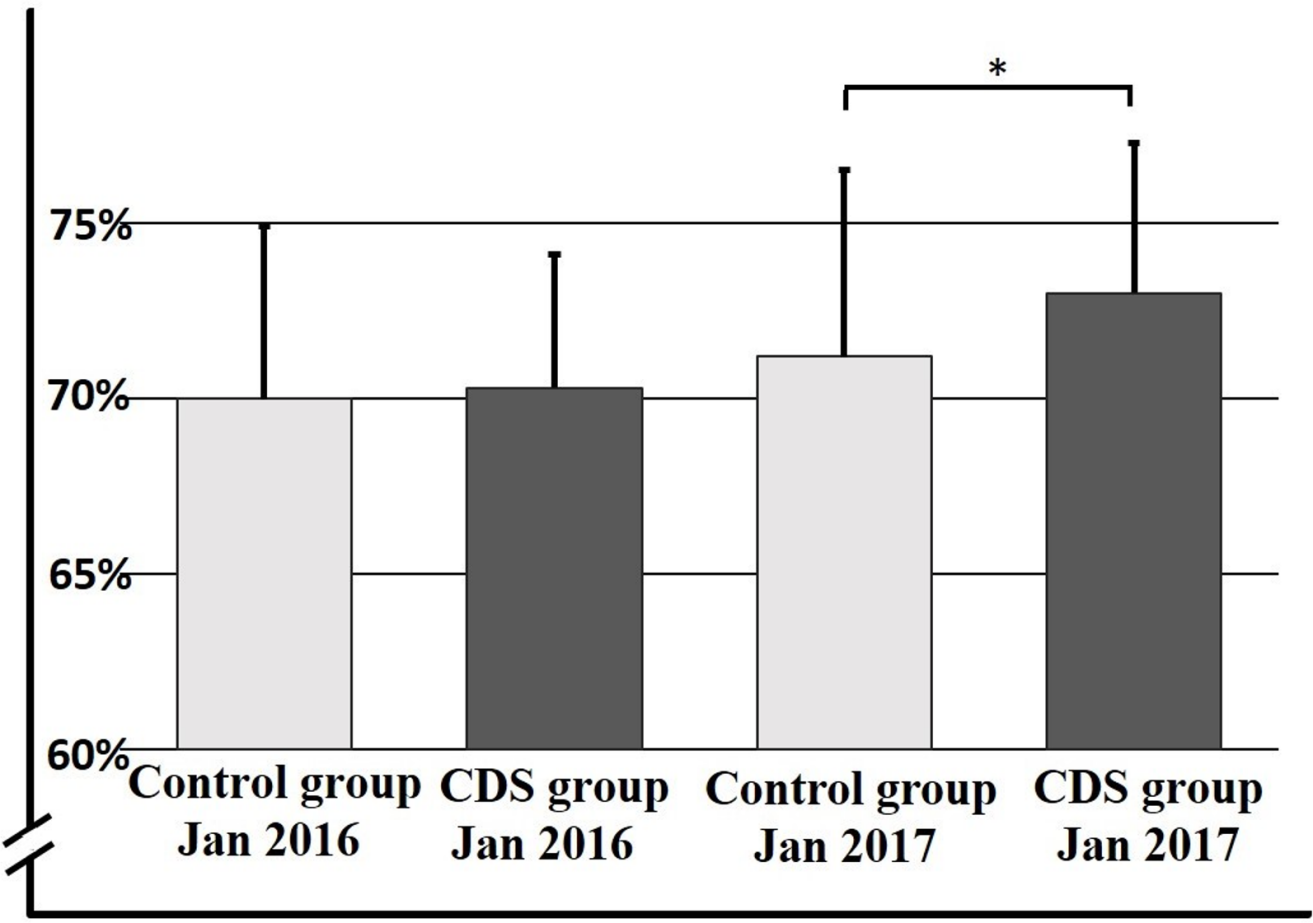
Proportion of eligible patients prescribed anticoagulant therapy after 12 months in control and CDS groups. Rate of adherence to guidelines presented as percentage on the *y*-axis, with the different study groups over time on the *x*-axis. Data are presented as mean ± SD. *p = 0.013. CDS, clinical decision support tool.

There was no difference in the baseline proportion of eligible patients prescribed anticoagulant therapy in the CDS and control groups (CDS group 70.3% [95% CI 62.9%–77.7%], control group 70.0% [95% CI 60.4%–79.6%], *p* = 0.83). After 12 months, there was a significant increase in anticoagulant prescription in the CDS versus the control group (73.0% [95% CI 64.6%–81.4%] versus 71.2% [95% CI 60.8%–81.6%], *p* = 0.013, with a treatment effect estimate of 0.016 [95% CI 0.003–0.028]; Fig 3; Table 2). Analysis revealed an intraclass correlation coefficient of 0.001. When we analyzed differences in rates of guideline adherence in different quartiles based on baseline rates, we could not show any difference between primary care clinics with a high and low baseline rate regarding impact from the CDS (first quartile range 0.61–0.69; fourth quartile range 0.76–0.80).

**Table 2:** Primary endpoint with adherence to guidelines at baseline and after 12 months.

Measure	January 2016		January 2017	
	Control group	CDS group	Control group	CDS group*
Adherence to guidelines (%)	70.0 (95% CI 62.9–77.7)	70.3 (95% CI 60.4–79.6)	71.2 (95% CI 60.8–81.6)	73.0 (95% CI 64.6–81.4)
Numerator (number of patients with AF adherent to guidelines)	4,187	5,186	4,346	5,734
Denominator (total number of patients with AF)**	6,009	7,370	6,156	7,861

*p = 0.013 versus control group in January 2017. Analysis with linear regression adjusted for baseline adherence to guidelines, and weighted by cluster size.

**Total number or patients with AF eligible for anticoagulant therapy based on CHA_2_DS_2_-VASc score.

AF, atrial fibrillation; CDS, clinical decision support tool.

### Stroke, TIA, and systemic thromboembolism

During the study period, the CDS group had an incidence of stroke, TIA, or systemic thromboembolism of 49 (95% CI 43–55) per 1,000 patients with AF compared to 47 (95% CI 39–55) per 1,000 patients with AF in the control group (*p* = 0.64).

### Safety outcome

During the study period, the CDS group had a lower incidence of significant bleeding, with 12 (95% CI 9–15) per 1,000 patients with AF compared to 16 (95% CI 12–20) per 1,000 patients with AF in the control group (*p* = 0.04).

### Reason for deviation from guidelines

**Fig 4:**
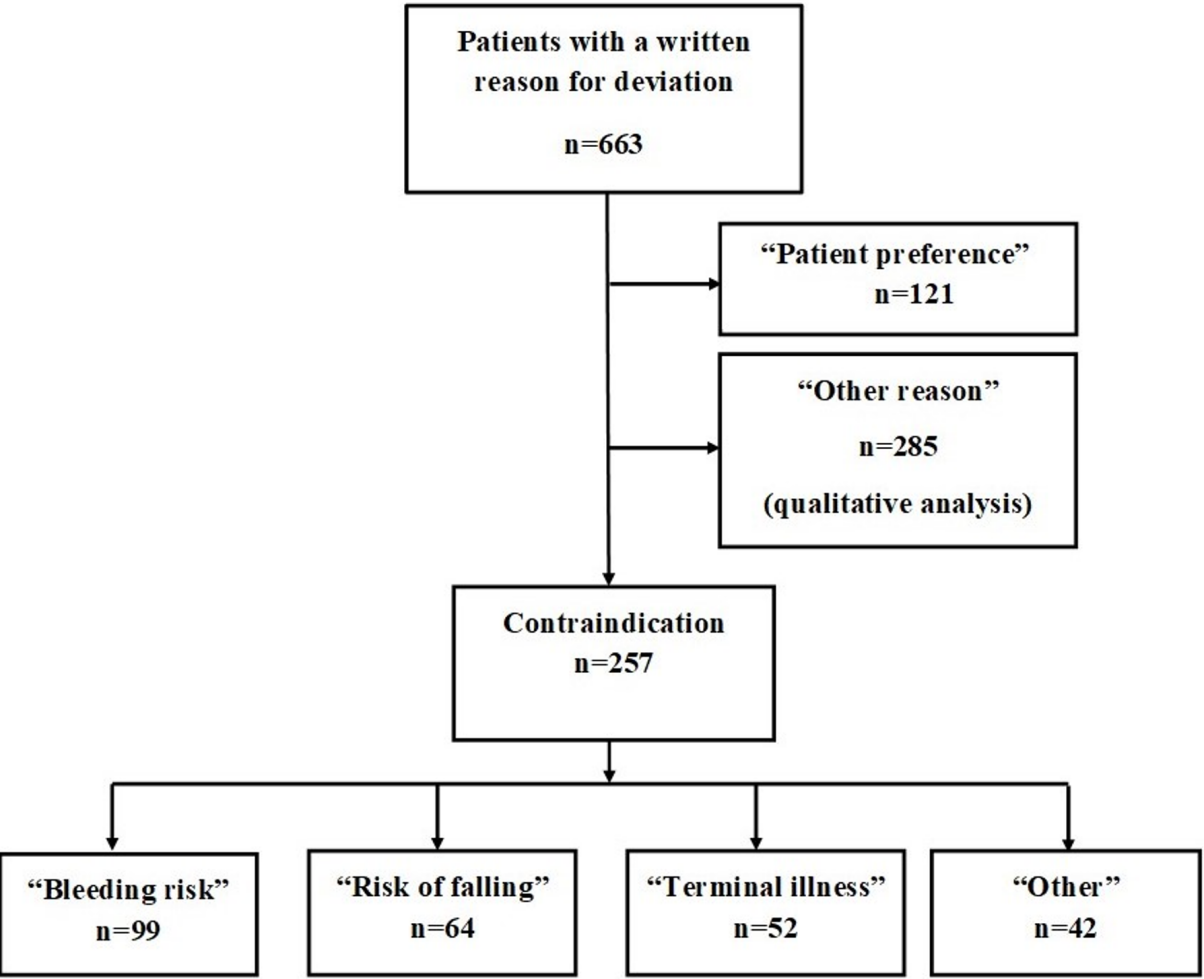
Flowchart of the specified reasons for not initiating anticoagulant therapy.

In 663 unique patients for whom guidelines indicated anticoagulant therapy, we were able to collect data regarding a specified reason why anticoagulant therapy was not initiated (Fig 4). Of the 663 written reasons for deviation from guidelines, 285 were stated as other reason and will undergo qualitative analysis and be published separately.

### Alert appearance

**Fig 5:**
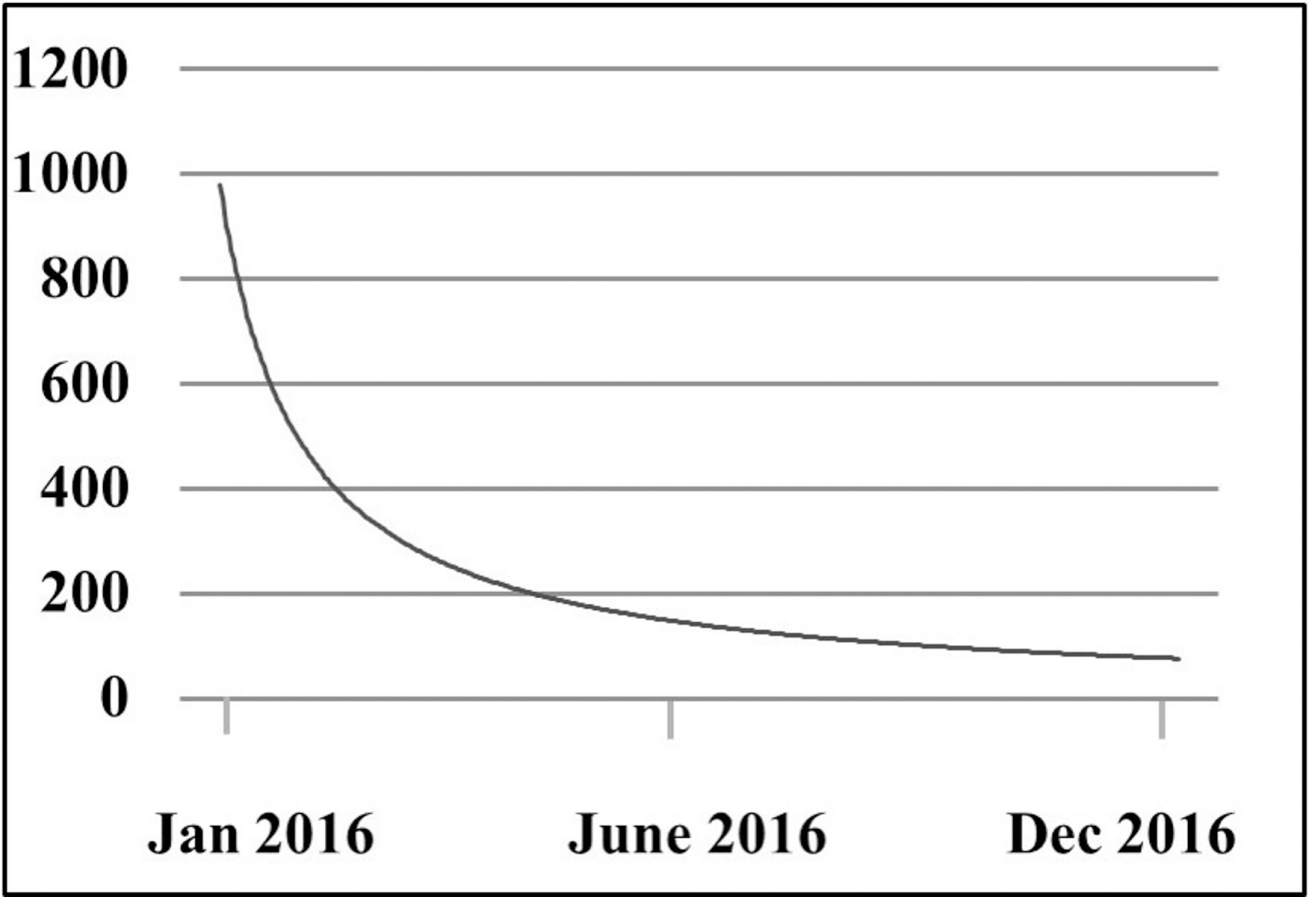
Number of times the clinical decision support alert was shown for the first time in unique individuals throughout the study period.

We analyzed the number of times the CDS appeared in the intervention group. By looking at the first time a CDS message was shown for a unique individual, we got an estimate of the frequency of the CDS alerts over time (Fig 5).

## Discussion

The present study demonstrates that a newly developed clinical decision support system yields a small increase in adherence to guidelines regarding stroke prevention in patients with AF. During the study period of 1 year, the intervention did not translate into a clinically significant reduction in stroke, TIA, and systemic thromboembolism. This study serves as a proof of concept that an easily available technique could increase preventive measures in healthcare.

The usage of clinical decision support systems is increasing. Data regarding long-term clinical outcome, however, are to some extent conflicting [14,15]. There is good evidence that clinical decision support systems can improve preventive measures. Kucher and colleagues showed that a clinical decision alert system could reduce the incidence of symptomatic and asymptomatic deep vein thrombosis among hospitalized patients, and other studies have shown this type of intervention to have sustained effects [25–27]. Furthermore, clinical decision support systems can improve patient safety, in particular by reducing medication errors [28,29]. On the other hand, evidence for beneficial outcome in other areas is sparse, and there is a lack of evidence for impact on mortality and health economy [18], with some reports indicating a potential for harm [30,31]. Furthermore, there is a need for further studies on workflow issues and the users’ notion of these systems [14]. Indeed, these issues have recently been addressed in the GUIDES project, with a structured checklist to support the design and implementation of computerized decision support tools [32].

AF is a common disorder with a proven benefit of anticoagulant therapy for the prevention of thromboembolic events [6]. Nevertheless, significant underuse of this therapy continues. Taken together, these factors make anticoagulant therapy in patients with AF a suitable target for a clinical decision support system in order to improve preventive measures. Until now, however, there were no data to our knowledge supporting this intervention in patients with AF. Cook et al. conducted a cohort study with historical controls for patients with new-onset AF and found no difference in physician prescription with their clinical decision support system [16]. Arts and colleagues conducted a randomized trial with no beneficial effect of their clinical decision support system, most likely due to lack of usage [19]. Recently, a large randomized trial in the primary care setting in the United Kingdom did not show any change in prescription pattern regarding anticoagulant use in patients with AF [20].

Indeed, there are potential reasons why the abovementioned systems did not work, including additional workload and the risk of false positive alerts—the latter contributing to the phenomenon known as “alert fatigue” [33]—as well as the risk of automation bias, where users tend to over-rely on computer output [34,35]. However, given the strong need for improvement of stroke prophylaxis in patients with AF, further development and evaluation of these complementary tools is relevant.

When designing a clinical decision support system, there are several factors to consider in order to increase the likelihood of a successful intervention. Kawamoto et al. present 4 clinical decision support features that contribute to improved clinical practice: “provide decision support automatically as part of clinician workflow,” “deliver decision support at the time and location of decision making,” “provide actionable recommendations,” and “use a computer to generate the decision support” [36]. When designing the current CDS, an attempt was made to fulfil all of these criteria. In accordance, an analysis regarding the users’ acceptance of the current intervention will be published separately, and hopefully will contribute to further understanding on how to best design these tools in order to enhance adherence to guidelines and effectiveness.

When discussing anticoagulant therapy in the AF population, it is important to recognize a circumstance that is specific to this issue: even though anticoagulant therapy is highly effective in preventing stroke in this population, not all patients with AF will benefit from the intervention. To date, we have no evidence that patients with a true low risk for stroke (CHA_2_DS_2_-VASc score of 0 if male or 1 if female) should receive therapy. Furthermore, in certain patients, the risks of anticoagulant therapy outweigh the benefits, such as patients with a high bleeding risk or patients unable to comply with the prescribed medication. The exact number of people who are eligible for therapy is therefore unknown. The Swedish National Board of Health and Welfare has postulated that 80% of patients with AF should be prescribed an anticoagulant agent [37]. Interestingly, in a recently published evaluation of a multifaceted intervention aiming to increase anticoagulant use, the intervention group reached a rate of adherence to guidelines of 80% [38]. The percentage postulated is further supported by data from the GLORIA-AF registry indicating this number as a reasonable goal [39]. In our study, the baseline adherence rate regarding anticoagulant treatment was high, but in line with a recent publication from the GARFIELD-AF registry indicating a steady increase in the prescription pattern since the introduction of NOACs [40]. With a postulated goal of 80%, the documented increase of anticoagulant use with our CDS intervention therefore represents an important step towards reaching this number.

A possible adverse effect with anticoagulant therapy is an increased incidence of bleeding. A clinical decision support system could potentially induce healthcare providers to initiate anticoagulant therapy in patients not suitable for this therapy. It is therefore encouraging that no increase in bleeding events was demonstrated in patients in the intervention group who initiated anticoagulant therapy during the study. Regarding the clinical outcome, we did not see any significant reduction in thromboembolic events in the intervention group. However, given the short observation time of 1 year, and the small increase in anticoagulant therapy usage, this is not surprising. A longer follow-up period would be required to show a possible beneficial effect of anticoagulant therapy on clinical events. Indeed, this analysis will be performed as per our study protocol 3 and 6 years after closure of the primary study period.

An important issue when trying to increase physicians’ adherence to guidelines for anticoagulant therapy is to understand the reasons why therapy is not initiated despite awareness of the AF diagnosis. When analyzing the frequency of the CDS alerts being shown, we noticed that the majority of the AF population attending their primary care facility received the intervention, as indicated by the steady decrease of CDS alerts in unique individuals throughout the study period. In contrast, the small increase in anticoagulant prescription in the CDS group indicates that in some cases the primary care physicians either ignored the recommendation or made a decision that the patient would not benefit from therapy. In light of this, it will be important to analyze the written reasons for not following the suggestion from the CDS.

Regarding the reasons stated as contraindication, the majority of patients did not receive treatment due to a combination of increased risk of bleeding and falls as well as terminal illness such as malignancy and dementia, in line with previous reports [41]. The prespecified “other reason” will be analyzed separately through qualitative analysis, and hopefully this analysis will enhance the understanding of the decision-making regarding this important issue.

There are a number of strengths of our study. Notably, we were able to include all primary care clinics in the county of Östergötland. Therefore, the current study covers the overall population of our catchment area. Furthermore, since no clinics declined to participate, we were able to reduce the likelihood of selection bias, where potentially primary care clinics already aware of this important issue would be more likely to participate.

There are several limitations of the current study. In our sample size calculation, we considered an increase of adherence from 70% to 75% clinically meaningful. We were not able to meet this level of increase, but still got a significant increase regarding adherence in our intervention group. In hindsight, given the high prevalence of AF and the proven benefit of anticoagulant therapy, we still consider our findings clinically meaningful, highlighting the fact that the definition of a clinically meaningful effect of any intervention in this setting is not well established, and requires further investigation. With the subset of patients with a new diagnosis of AF during the study, differential recruitment represents another possible limitation. Furthermore, this group of patients had significant differences regarding baseline characteristics compared to the population of patients included at baseline. We were, however, not able to analyze if there was a difference regarding the effect of our intervention in the population with a new diagnosis of AF as compared to the population included at baseline. Additionally, the study was conducted in a region with publicly funded healthcare. Therefore, it might be difficult to extrapolate our findings to countries with alternative healthcare and reimbursement systems. With our study carried out in a region with a high baseline rate regarding adherence to guidelines, it is less clear what benefit the intervention would provide with different baseline rates. However, the argument can be made that these interventions would have a greater potential benefit where adherence to guidelines is worse. Furthermore, we were not able to demonstrate any clinical benefit regarding a reduction of clinical events during our primary study period. However, the net clinical benefit of anticoagulant therapy has been shown in previous studies [6], and we will monitor for a late beneficial effect regarding stroke, TIA, and systemic thromboembolism. Finally, we experienced a closedown of 1 of the primary care clinics in the control group, which led to a crossover effect.

In conclusion, the current study shows that a newly developed clinical decision support system in the primary care setting contributed to a small improvement regarding anticoagulant therapy in patients with AF. This easily implementable intervention represents an additional tool for stroke prevention measures in the AF population, and highlights the feasibility of using a CDS to enhance patient care in other areas of medicine.

## Supporting information

S1 CONSORT Checklist (DOCX)

S1 Data (XLSX)

S1 Text ICD and ATC codes. (DOCX)

## Abbreviations

AF: atrial fibrillation
CDS: clinical decision support tool
EHR: electronic health record
ICD: International Classification of Diseases
NOAC: non–vitamin K oral anticoagulant
TIA: transient ischemic attack

## References

[1] Friberg L, Bergfeldt L. Atrial fibrillation prevalence revisited. J Intern Med. 2013;274(5):461–8. doi: 10.1111/joim.12114 23879838 10.1111/joim.12114

[2] Chugh SS, Havmoeller R, Narayanan K, Singh D, Rienstra M, Benjamin EJ, et al Worldwide epidemiology of atrial fibrillation: a Global Burden of Disease 2010 Study. Circulation. 2014;129(8):837–47. doi: 10.1161/CIRCULATIONAHA.113.005119 24345399 10.1161/CIRCULATIONAHA.113.005119 PMC4151302

[3] Wendelboe AM, Raskob GE. Global burden of thrombosis: epidemiologic aspects. Circ Res. 2016;118(9):1340–7. doi: 10.1161/CIRCRESAHA.115.306841 27126645 10.1161/CIRCRESAHA.115.306841

[4] Andrade J, Khairy P, Dobrev D, Nattel S. The clinical profile and pathophysiology of atrial fibrillation: relationships among clinical features, epidemiology, and mechanisms. Circ Res. 2014;114(9):1453–68. doi: 10.1161/CIRCRESAHA.114.303211 24763464 10.1161/CIRCRESAHA.114.303211

[5] Gladstone DJ, Bui E, Fang J, Laupacis A, Lindsay MP, Tu JV, et al Potentially preventable strokes in high-risk patients with atrial fibrillation who are not adequately anticoagulated. Stroke. 2009;40(1):235–40. doi: 10.1161/STROKEAHA.108.516344 18757287 10.1161/STROKEAHA.108.516344

[6] Hart RG, Pearce LA, Aguilar MI. Meta-analysis: antithrombotic therapy to prevent stroke in patients who have nonvalvular atrial fibrillation. Ann Intern Med. 2007;146(12):857–67. 17577005 10.7326/0003-4819-146-12-200706190-00007

[7] Camm AJ, Lip GY, De Caterina R, Savelieva I, Atar D, Hohnloser SH, et al 2012 focused update of the ESC Guidelines for the management of atrial fibrillation: an update of the 2010 ESC Guidelines for the management of atrial fibrillation—developed with the special contribution of the European Heart Rhythm Association. Europace. 2012;14(10):1385–413. doi: 10.1093/europace/eus305 22923145 10.1093/europace/eus305

[8] Lip GY, Nieuwlaat R, Pisters R, Lane DA, Crijns HJ. Refining clinical risk stratification for predicting stroke and thromboembolism in atrial fibrillation using a novel risk factor-based approach: the euro heart survey on atrial fibrillation. Chest. 2010;137(2):263–72. doi: 10.1378/chest.09-1584 19762550 10.1378/chest.09-1584

[9] Bjorck S, Palaszewski B, Friberg L, Bergfeldt L. Atrial fibrillation, stroke risk, and warfarin therapy revisited: a population-based study. Stroke. 2013;44(11):3103–8. doi: 10.1161/STROKEAHA.113.002329 23982711 10.1161/STROKEAHA.113.002329

[10] Kakkar AK, Mueller I, Bassand JP, Fitzmaurice DA, Goldhaber SZ, Goto S, et al Risk profiles and antithrombotic treatment of patients newly diagnosed with atrial fibrillation at risk of stroke: perspectives from the international, observational, prospective GARFIELD registry. PLoS ONE. 2013; 8(5):e63479 doi: 10.1371/journal.pone.0063479 23704912 10.1371/journal.pone.0063479 PMC3660389

[11] Ogilvie IM, Newton N, Welner SA, Cowell W, Lip GY. Underuse of oral anticoagulants in atrial fibrillation: a systematic review. Am J Med. 2010;123(7):638–45 e4. doi: 10.1016/j.amjmed.2009.11.025 20609686 10.1016/j.amjmed.2009.11.025

[12] Xian Y, O’Brien EC, Liang L, Xu H, Schwamm LH, Fonarow GC, et al Association of preceding antithrombotic treatment with acute ischemic stroke severity and in-hospital outcomes among patients with atrial fibrillation. JAMA. 2017;317(10):1057–67. doi: 10.1001/jama.2017.1371 28291892 10.1001/jama.2017.1371

[13] Oldgren J, Healey JS, Ezekowitz M, Commerford P, Avezum A, Pais P, et al Variations in cause and management of atrial fibrillation in a prospective registry of 15,400 emergency department patients in 46 countries: the RE-LY Atrial Fibrillation Registry. Circulation. 2014;129(15):1568–76. doi: 10.1161/CIRCULATIONAHA.113.005451 24463370 10.1161/CIRCULATIONAHA.113.005451

[14] Bright TJ, Wong A, Dhurjati R, Bristow E, Bastian L, Coeytaux RR, et al Effect of clinical decision-support systems: a systematic review. Ann Intern Med. 2012;157(1):29–43. doi: 10.7326/0003-4819-157-1-201207030-00450 22751758 10.7326/0003-4819-157-1-201207030-00450

[15] Lobach D, Sanders GD, Bright TJ, Wong A, Dhurjati R, Bristow E, et al Enabling health care decisionmaking through clinical decision support and knowledge management. Evid Rep Technol Assess (Full Rep). 2012;(203):1–784. PMC4781172 23126650

[16] Cook DA, Enders F, Caraballo PJ, Nishimura RA, Lloyd FJ. An automated clinical alert system for newly-diagnosed atrial fibrillation. PLoS ONE. 2015; 10(4):e0122153 doi: 10.1371/journal.pone.0122153 25849969 10.1371/journal.pone.0122153 PMC4388495

[17] Holt TA, Thorogood M, Griffiths F. Changing clinical practice through patient specific reminders available at the time of the clinical encounter: systematic review and meta-analysis. J Gen Intern Med. 2012;27(8):974–84. doi: 10.1007/s11606-012-2025-5 22407585 10.1007/s11606-012-2025-5 PMC3403145

[18] Moja L, Kwag KH, Lytras T, Bertizzolo L, Brandt L, Pecoraro V, et al Effectiveness of computerized decision support systems linked to electronic health records: a systematic review and meta-analysis. Am J Public Health. 2014;104(12):e12–22. doi: 10.2105/AJPH.2014.302164 25322302 10.2105/AJPH.2014.302164 PMC4232126

[19] Arts DL, Abu-Hanna A, Medlock SK, van Weert HC. Effectiveness and usage of a decision support system to improve stroke prevention in general practice: a cluster randomized controlled trial. PLoS ONE. 2017; 12(2):e0170974 doi: 10.1371/journal.pone.0170974 28245247 10.1371/journal.pone.0170974 PMC5330455

[20] Holt TA, Dalton A, Marshall T, Fay M, Qureshi N, Kirkpatrick S, et al Automated software system to promote anticoagulation and reduce stroke risk: cluster-randomized controlled trial. Stroke. 2017;48(3):787–90. doi: 10.1161/STROKEAHA.116.015468 28119433 10.1161/STROKEAHA.116.015468 PMC5351848

[21] Karlsson LO, Nilsson S, Charitakis E, Bang M, Johansson G, Nilsson L, et al Clinical decision support for stroke prevention in atrial fibrillation (CDS-AF): rationale and design of a cluster randomized trial in the primary care setting. Am Heart J. 2017;187:45–52. doi: 10.1016/j.ahj.2017.02.009 28454807 10.1016/j.ahj.2017.02.009

[22] Hemming K, Girling AJ, Sitch AJ, Marsh J, Lilford RJ. Sample size calculations for cluster randomised controlled trials with a fixed number of clusters. BMC Med Res Methodol. 2011;11:102 doi: 10.1186/1471-2288-11-102 21718530 10.1186/1471-2288-11-102 PMC3149598

[23] Campbell MK, Mollison J, Steen N, Grimshaw JM, Eccles M. Analysis of cluster randomized trials in primary care: a practical approach. Fam Pract. 2000;17(2):192–6. 10758085 10.1093/fampra/17.2.192

[24] Shrout PE, Fleiss JL. Intraclass correlations: uses in assessing rater reliability. Psychol Bull. 1979;86(2):420–8. 18839484 10.1037//0033-2909.86.2.420

[25] Beeler PE, Kucher N, Blaser J. Sustained impact of electronic alerts on rate of prophylaxis against venous thromboembolism. Thromb Haemost. 2011;106(4):734–8. doi: 10.1160/TH11-04-0220 21800010 10.1160/TH11-04-0220

[26] Kucher N, Koo S, Quiroz R, Cooper JM, Paterno MD, Soukonnikov B, et al Electronic alerts to prevent venous thromboembolism among hospitalized patients. N Engl J Med. 2005;352(10):969–77. doi: 10.1056/NEJMoa041533 15758007 10.1056/NEJMoa041533

[27] Lecumberri R, Marques M, Diaz-Navarlaz MT, Panizo E, Toledo J, Garcia-Mouriz A, et al Maintained effectiveness of an electronic alert system to prevent venous thromboembolism among hospitalized patients. Thromb Haemost. 2008;100(4):699–704. 18841295 10.1160/th08-05-0337

[28] Kaushal R, Shojania KG, Bates DW. Effects of computerized physician order entry and clinical decision support systems on medication safety: a systematic review. Arch Intern Med. 2003;163(12):1409–16. doi: 10.1001/archinte.163.12.1409 12824090 10.1001/archinte.163.12.1409

[29] Wolfstadt JI, Gurwitz JH, Field TS, Lee M, Kalkar S, Wu W, et al The effect of computerized physician order entry with clinical decision support on the rates of adverse drug events: a systematic review. J Gen Intern Med. 2008;23(4):451–8. doi: 10.1007/s11606-008-0504-5 18373144 10.1007/s11606-008-0504-5 PMC2359507

[30] Coiera E, Westbrook J, Wyatt J. The safety and quality of decision support systems. Yearb Med Inform. 2006:20–5. 17051290

[31] Myers RB, Jones SL, Sittig DF. Review of reported clinical information system adverse events in US Food and Drug Administration databases. Appl Clin Inform. 2011;2(1):63–74. doi: 10.4338/ACI-2010-11-RA-0064 21938265 10.4338/ACI-2010-11-RA-0064 PMC3175794

[32] Van de Velde S, Aertgeerts B, Flottorp S, Kortteisto T, Kunnamo I, Roshanov P, et al GUIDES checklist: a tool to assist professionals when implementing guidelines with computerised decision support (CDS). Oslo: Norwegian Institute of Public Health; 2018 [cited 2018 Feb 15]. Available from: https://www.guidesproject.org/.

[33] van der Sijs H, Aarts J, Vulto A, Berg M. Overriding of drug safety alerts in computerized physician order entry. J Am Med Inform Assoc. 2006;13(2):138–47. doi: 10.1197/jamia.M1809 16357358 10.1197/jamia.M1809 PMC1447540

[34] Ash JS, Sittig DF, Campbell EM, Guappone KP, Dykstra RH. Some unintended consequences of clinical decision support systems. AMIA Annu Symp Proc. 2007:26–30. 18693791 PMC2813668

[35] Goddard K, Roudsari A, Wyatt JC. Automation bias—a hidden issue for clinical decision support system use. Stud Health Technol Inform. 2011;164:17–22. 21335682

[36] Kawamoto K, Houlihan CA, Balas EA, Lobach DF. Improving clinical practice using clinical decision support systems: a systematic review of trials to identify features critical to success. BMJ. 2005;330(7494):765 doi: 10.1136/bmj.38398.500764.8F 15767266 10.1136/bmj.38398.500764.8F PMC555881

[37] National Board of Health and Welfare. National guidelines for cardiac care. Stockholm: National Board of Health and Welfare; 2015.

[38] Vinereanu D, Lopes RD, Bahit MC, Xavier D, Jiang J, Al-Khalidi HR, et al A multifaceted intervention to improve treatment with oral anticoagulants in atrial fibrillation (IMPACT-AF): an international, cluster-randomised trial. Lancet. 2017;390(10104):1737–46. doi: 10.1016/S0140-6736(17)32165-7 28859942 10.1016/S0140-6736(17)32165-7

[39] Huisman MV, Rothman KJ, Paquette M, Teutsch C, Diener HC, Dubner SJ, et al The changing landscape for stroke prevention in AF: findings from the GLORIA-AF registry phase 2. J Am Coll Cardiol. 2017;69(7):777–85. doi: 10.1016/j.jacc.2016.11.061 28209218 10.1016/j.jacc.2016.11.061

[40] Camm AJ, Accetta G, Ambrosio G, Atar D, Bassand JP, Berge E, et al Evolving antithrombotic treatment patterns for patients with newly diagnosed atrial fibrillation. Heart. 2017;103(4):307–14. doi: 10.1136/heartjnl-2016-309832 27647168 10.1136/heartjnl-2016-309832 PMC5293840

[41] Pugh D, Pugh J, Mead GE. Attitudes of physicians regarding anticoagulation for atrial fibrillation: a systematic review. Age Ageing. 2011;40(6):675–83. doi: 10.1093/ageing/afr097 21821732 10.1093/ageing/afr097

