# Supplementary material for: A clinical decision support tool for improving adherence to guidelines on anticoagulant therapy in patients with atrial fibrillation at risk of stroke: A cluster-randomized trial in a Swedish primary care setting (the CDS-AF study)
Source: PLoS Med. 2018 Mar 13;15(3):e1002528. doi: 10.1371/journal.pmed.1002528 (PMC5849292; doi:10.1371/journal.pmed.1002528)
Supplement: S1 Text — (DOCX) [file pmed.1002528.s003.docx]

**S1 Text.** **ICD and ATC codes**

**ICD codes for the CHA_2_DS_2_VASc algorithm:**

I50 Heart failure

I10-15 Hypertension

E10-14 Diabetes mellitus

I21, I252, I70-73 Vascular disease

I63-64, G45, I74 Thromboembolic event

**ICD codes for significant bleeding**

I60-62, K25-28 (subcodes 0,2,4,6 only), I85.0, I98.3, K62.5, K92.2, D62.9

**ICD code for chronic kidney disease**

N18

**ATC codes for anticoagulant therapy**

B01AE07 Dabigatran

B01AA03 Warfarin

B01AF02 Apixaban

B01AF01 Rivaroxaban
